# Linking the wintering and breeding grounds of warblers along the Pacific Flyway

**DOI:** 10.1002/ece3.3222

**Published:** 2017-07-18

**Authors:** David P. L. Toews, Julian Heavyside, Darren E. Irwin

**Affiliations:** ^1^ Department of Zoology and Biodiversity Research Centre University of British Columbia Vancouver BC Canada; ^2^ Cornell Lab of Ornithology Cornell University Ithaca NY USA

**Keywords:** Migration, isotopes, connectivity, wood warbler

## Abstract

Long‐distance migration is a behavior that is exhibited by many animal groups. The evolution of novel migration routes can play an important role in range expansions, ecological interactions, and speciation. New migration routes may evolve in response to selection in favor of reducing distance between breeding and wintering areas, or avoiding navigational barriers. Many migratory changes are likely to evolve gradually and are therefore difficult to study. Here, we attempt to connect breeding and wintering populations of myrtle warblers (*Setophaga coronata coronata*) to better understand the possible evolution of distinct migration routes within this species. Myrtle warblers, unlike most other warblers with breeding ranges primarily in eastern North America, have two disjunct overwintering concentrations—one in the southeastern USA and one along the Pacific Coast—and presumably distinct routes to‐and‐from these locations. We studied both myrtle and Audubon's warblers (*S. c. auduboni*) captured during their spring migration along the Pacific Coast, south of the narrow region where these two taxa hybridize. Using stable hydrogen isotopes and biometric data, we show that those myrtle warblers wintering along the southern Pacific Coast of North America are likely to breed at high latitudes in Alaska and the Yukon rather than in Alberta or further east. Our interpretation is that the evolution of this wintering range and migration route along the Pacific Coast may have facilitated the breeding expansion of myrtle warblers into northwestern North America. Moreover, these data suggest that there may be a migratory divide within genetically similar populations of myrtle warblers.

## 1 INTRODUCTION

Many migrating animals travel great distances between their wintering and breeding grounds. In many species, the process of migrating between these distant areas is both a navigational and energetic challenge. This has resulted in the evolution of finely tuned migration phenotypes—particularly well‐studied in avian systems—that are the product of adaptive evolution (Alerstam, 1990). By extension, the constraints imposed by a long migration have also shaped the range limits of the breeding and wintering grounds of migratory species and, specifically, limited range expansions of some taxa (Bensch, 1999; Bohning‐Gaese, Gonzalez‐Guzman, & Brown, 1998; Irwin & Irwin, 2005; Toews, 2017). For instance, Toews (2017) used niche models to identify regions in high latitude areas of North America that were not occupied by several wood warbler species but were, in fact, identified as suitable habitat. The discordance between these species’ fundamental and realized niches is likely due in part to challenges associated with migrating from their wintering areas to these remote regions.

Toews (2017) suggested two scenarios that might reduce the costs of migration and facilitate the exploitation of these distant, yet presumably suitable habitats. First, taxa might, over time, evolve adaptations that increase efficiency and enable longer migratory routes. Blackpoll warblers (*Setophaga striata*), for example, execute an extraordinary, nonstop, transoceanic migration that connects their breeding range, which extends as far north as northern Alaska, to their wintering range in South America (DeLuca et al., 2015). Blackpoll warblers have likely evolved a suite of physiological, neurological, and morphological adaptations (e.g., Hussell & Lambert, 1980) that allowed them to extend their long migration as they expanded northwestward into Alaska following glacial retreat.

A second possibility to allow for breeding range expansion is for a species to expand or change its overwintering area or change its migratory route. These shifts might lessen the distance between wintering and breeding areas or avoid navigational barriers, like large lakes or mountain ranges. However, large shifts in migratory routes and/or wintering locations appear to be evolutionarily difficult. For example, the northern wheatear (*Oenanthe oenanthe*) is the only Old World thrush in its genus to have colonized North America. Isotopic and geolocator data demonstrate that wheatears in the New World have retained their ancestral sub‐Saharan wintering grounds, resulting in a remarkable cross‐hemisphere migration (Bairlein et al., 2012). Shifts in migration routes are even more likely to be challenging if intermediate routes would take migrants across regions that are ecologically inferior or difficult to navigate across (Bensch, Andersson, & Akesson, 1999; Irwin, 2009; Irwin & Irwin, 2005); this may explain the rarity of expansions of long‐distance migrants between Eurasia and North America.

Range and migration changes in high latitude species must have been substantial over the past several million years, particularly in response to the glaciations during the Pleistocene (Ruegg & Smith, 2002; Weir & Schluter, 2004). However, to study range and migratory changes in an evolutionary context, such shifts must either occur rapidly and over the period of study (e.g., the contemporary overwintering expansion of blackcap warblers, *Sylvia atricapilla*, into Britain and Ireland; Berthold & Terrill, 1988; Bearhop et al., 2005; Irwin, 2009) or biogeographic patterns must lead to a strong inference of evolutionary history. Here, we use the latter approach and take advantage of the distinctive breeding and overwintering patterns exhibited by the myrtle warbler (*Setophaga coronata coronata*; here we follow the taxonomic treatment of North American Nomenclature Committee, which treats the myrtle warbler as a subspecies of the yellow‐rumped warbler species; it should be noted however that the IOC World Bird List treats myrtle warblers as a species distinct from other forms in the yellow‐rumped warbler species comple*x*).

**Figure 1:**
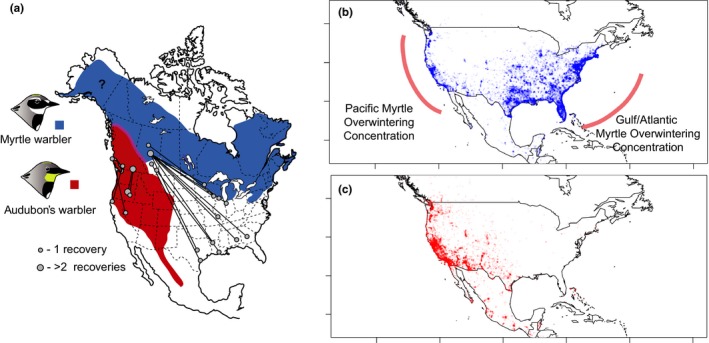
(a) Distribution and band recoveries of Audubon's and myrtle warblers. Banding data obtained from Brewer, Diamond, Woodsworth, Collins, and Dunn (2006) and the Canadian Bird Banding Office (2013). There is a hybrid zone between Audubon's and myrtle warblers where their ranges come into contact in western Canada. Overwintering records of myrtle (b) and Audubon's (c) warblers, up to 2015, retrieved from eBird (Sullivan et al., 2009). Records include only sightings between November and March. The two myrtle warbler overwintering concentrations (Pacific Coast and Gulf/Atlantic) are highlighted in on the map. The majority of Audubon's warblers winter along the Pacific Coast and Mexico

Myrtle warblers exhibit several notable characteristics in terms of their breeding and wintering ranges. First, they are one of the few eastern warblers that have breeding ranges that are both mostly restricted to the boreal forest and also breed far into Alaska and the Yukon (Figure 1a; biogeographic pattern similar to Figure 2c in Toews, 2017). This contrasts with most other eastern boreal wood warblers, which have ranges that do not extend as far into the northwest. Second, myrtle warblers have two distinct overwintering concentrations in the southern USA: One concentration of wintering myrtle warblers occurs along the Gulf coast; the second is along the Pacific Coast of California and Oregon (Figure 1b). Third, myrtle warblers hybridize extensively with their western relative, the Audubon's warbler (*S. c. auduboni;* currently treated by the North American Nomenclature Committee as a subspecies of the yellow‐rumped warbler species), in a narrow hybrid zone running north–south through the Canadian Rockies and then east–west across British Columbia to the Pacific Coast (Figure 1a; Hubbard, 1969; Barrowclough, 1980; Brelsford & Irwin, 2009). Audubon's warblers winter along the Pacific Coast of California and Mexico (Figure 1c).

The wintering myrtle warblers along the Pacific Coast have, by some authorities, been classified as a distinct subspecies of myrtle warbler—*S. coronata hooveri* (McGregor, 1899)—distinguishing it from the more widespread eastern form *S. c. coronata*. Early work found these warblers “in colors and markings like *[Setophaga c.] coronata* but with wing[s] and tail[s] much longer” (McGregor, 1899). However, given the subtle differences in plumage and morphology, some authors have questioned the validity of such a “finely drawn subspecies” (Bent, 1953; Hubbard, 1970; Yarbrough & Johnston, 1965). In the original description, *hooveri* from coastal California was presumed to breed in Alaska and northern British Columbia (McGregor, 1899). Subsequent work has found that northern myrtle warblers—within the presumed breeding range of *hooveri*—do have longer wings, on average (Hubbard, 1970). However, there have been no formal tests or direct evidence of connections between breeding and wintering populations.

There are many myrtle warblers that move through the lower mainland of coastal British Columbia during fall and spring migration, which is a region that is well within the breeding range of Audubon's rather than myrtle warblers. It is possible that these are those that winter along the Pacific Coast, providing us an opportunity to study the characteristics of this specific group. Here, we use morphological and isotopic analyses (Bowen, Liu, Vander Zanden, Zhao, & Takahashi, 2014; Hobson, 1999; Hobson, Van Wilgenburg, Wassenaar, & Larson, 2012; Kelly, Atudorei, Sharp, & Finch, 2002; Paxton, Yau, Moore, & Irwin, 2013; Toews, Brelsford, & Irwin, 2014) to infer the breeding region of these myrtle warblers on migration. We contrast this isotopic variation with Audubon's warblers migrating at the same time, but have a more restricted range of possible breeding locations within the southern half of British Columbia (e.g., Figure 1a) and are therefore used to indirectly validate our isotopic analysis.

For the breeding location of myrtle warblers, we distinguish between two possibilities. First, these birds could be primarily from the far northwestern breeding region of myrtle warblers, as is postulated for the range of *hooveri* (e.g., the Yukon and Alaska). Alternatively, these myrtle warblers could be from Alberta or other more eastern parts of the range of the *coronata* form, or they could be a broad mixture of *coronata* and *hooveri*. A finding that the western‐migrating myrtle warblers are primarily from the Yukon/Alaska region would raise the possibility of the *hooveri* form having evolved a distinct migratory route compared to the *coronata* form, suggesting the possibility of a migratory divide between two forms of myrtle warblers (Helbig, 1991; Bensch et al., 1999; Irwin & Irwin 2005; Ruegg, 2008; Rohwer & Irwin, 2011; Delmore & Irwin, 2014).

## 2 METHODS

### 2.1 Sampling, measurements, and feather stable isotopes

We temporarily captured myrtle and Audubon's warblers between April 18th and May 10th in 2014 at the Iona Island Bird Observatory, located in a riparian habitat at the Fraser River delta in southern British Columbia. Over the 2014 spring migration, 311 Audubon's and 235 myrtle warblers were banded at the observatory (WildResearch 2014). We focused the sampling for the current analysis across 6 days during spring migration (April 19–21st, 23rd, 27th, and 28th) when there were large numbers of warblers moving through the region. We determined the age and sex of each individual, and classified each—based on plumage—as Audubon's, myrtle, or hybrids (although we only identified a single hybrid). We took photographs of each bird and measured several morphometric traits, reporting wing chord and tail length here. We focused our analysis on male birds only, as they are the most confidently classified to species based on plumage. To compare the warblers to measurements from individuals across the range, we used data collected by Brelsford and Irwin (2009) as well as Hubbard (1970).

For a subset of Iona Island male warblers, we determined the stable hydrogen ratio (δ^2^Hf) in their covert feathers (*n *=* *59 individuals, divided approximately equally between myrtle, *n *=* *30, and Audubon's warblers, *n *=* *29) sampled across the migratory period. Stable hydrogen ratio here refers to the relative amounts of the two stable forms of hydrogen (deuterium over protium) divided by that ratio in a standard material. We call this ratio of ratios the “isotope value” of the feather. We took advantage of a distinctive pattern in the molt cycle of these warblers: In the fall, each bird molts all of its feathers during a prebasic molt, which takes place on the breeding grounds (Pyle, 1997). Prior to spring migration, these birds again molt three to four of their inner greater covert feathers on their wintering grounds during their prealternate molt (Gaddis, 2011). Therefore, on any single individual caught during the spring, there are two generations of feathers that can be easily distinguished visually (by the extent of white edging): one set with the isotopic values of the previous breeding ground and another from the most recent wintering area. For all but seven individuals, we analyzed paired isotope data (i.e., both basic and alternate feathers from the same individual), resulting in *n *=* *111 feathers with associated hydrogen data. Isotope analysis was carried out at Cornell University's stable isotope laboratory, and isotope corrections were performed using established keratin standards. The samples were run over 2 days. Information on international standards (KHS and CBS), as well as internal keratin standards, is reported in Table S1 (across the sample run the standard deviation of the internal keratin sample was 2‰). Experimental samples were weighed to an average of 0.848 mg (±0.005 *SD*). Hydrogen isotope values are reported as the corrected ^2^H value measured against the Vienna Standard Mean Ocean Water (i.e., δ^2^H_VSMOW_). To compare isotope values between myrtle and Audubon's warblers, we used two‐sample *t* tests as implemented in R 3.4.0 (R Core Team 2017). We also combined isotope and morphometric data to better assign individuals to specific breeding populations. For this, we used a linear regression between wing‐plus‐tail measures and the hydrogen value of the feathers, using the “lm” function in R.

### 2.2 IsoMAP analysis

We estimated the geographic origin of the feathers using IsoMAP, which is a framework that allows for modeling, predicting, and analysis of “isoscapes” (Bowen et al., 2014; http://www.isomap.org, accessed 4‐6‐2015). Stable hydrogen ratios correlate strongly with precipitation and vary, at a broad scale, by latitude (with higher latitudes having less deuterium; Meehan, Giermakowski, & Cryan, 2004; Hobson et al., 2012), although there is much local variation that relates to other biotic and abiotic differences, such as elevation. We employed the same geographic assignment approach as outlined in Toews et al. (2014). In this study, we used a precipitation hydrogen model within a longitudinal range of 168.4° to 51°W and a latitudinal range of 16.6° to 71.5°N (IsoMAP jobkey: 46203).

There is not a 1:1 relationship between hydrogen in precipitation (δ^2^Hp) and hydrogen in feathers (δ^2^Hf). For organic samples, such as feather keratin (δ^2^Hf), it is therefore important to generate an empirically based transfer function between the two (Bowen et al., 2014). We used the two‐part linear transfer function from Toews et al. (2014), which was modified from Hobson et al. (2012) and is based on hydrogen isotope values from passerine feathers grown at known locations. For feathers with δ^2^Hf below −53.6 ‰, we used δ^2^Hf = 0.5765*δ^2^Hp‐61.34 as the transfer function; for higher values, we used δ^2^Hf = 1.345*δ^2^Hp‐20.17. With IsoMAP, we then generated a geographic likelihood assignment surface for each feather using the “individual assignment” function, including the standard deviation of the residuals from the water/feather transformation function (9.96‰). The resulting likelihood surfaces were then averaged across individuals—separated by species and feather type—using the raster calculator in QGIS (QGIS Development Team 2017).

## 3 RESULTS

Consistent with observations from previous years, a pulse of myrtle and Audubon's warblers moved through southern British Columbia during a short period between the middle and end of April (approximately 500 individuals of both species over 3 weeks were banded at the Iona Island Bird Observatory). The Audubon's warblers we sampled during this time had wing chords similar to other Audubon's warblers measured throughout the interior of British Columbia (Table 1). The wings of migrant myrtle warblers were relatively long and were similar to the high latitude breeding populations of *hooveri* (Table 1), measuring approximately 2 mm longer than myrtle warblers sampled at lower latitudes from across their range (Table 1).

**Table 1:** Wing chord measures of myrtle and Audubon's warblers sampled previously by Hubbard (1970) and for the current study (captured on Iona Island, birds on migration in southern British Columbia). Audubon's warblers have similar wing lengths as those sampled throughout B.C.; Myrtle warblers from the current study have wing lengths similar to the *hooveri* subspecies, as measured by Hubbard (1970), and are much longer than birds sampled from Alberta eastward

Taxon	Season	Sample	Wing chord (mm)	*SD*	Sample size	Reference
Audubon's	Migrant	Iona Island, B.C.	76.3	2.3	26	This study
Audubon's	Breeding	Central B.C.	76.5	1.3	10	Hubbard (1970)
Myrtle	Migrant	Iona Island, B.C.	76.4	1.6		This study
Myrtle (*hooveri*)	Breeding	Alaska	77.1	1.9	14	Hubbard (1970)
Myrtle (*hooveri*)	Breeding	Yukon	76.5	1.6	13	Hubbard (1970)
Myrtle (*hooveri*)	Breeding	Northwest B.C.	76.8	1.1	13	Hubbard (1970)
Myrtle (*coronata*)	Breeding	Alberta	74.1	1.6	8	Hubbard (1970)
Myrtle (*coronata*)	Breeding	Manitoba	73.0	—	8	Hubbard (1970)
Myrtle (*coronata*)	Breeding	Newfoundland	74.7	2.4	6	Hubbard (1970)

**Figure 2:**
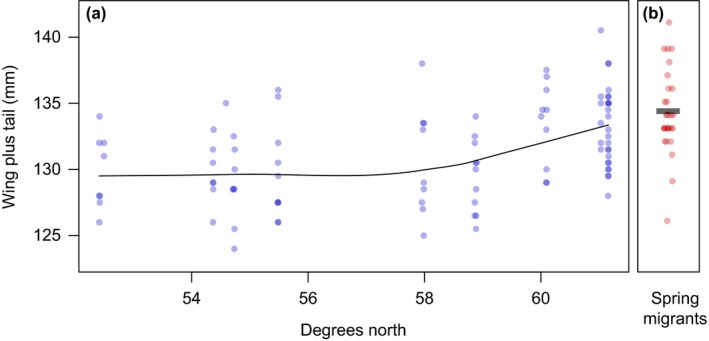
Morphometric analysis of myrtle warblers (a) sampled previously (blue points, from Brelsford & Irwin, 2009) and (b) from the current study (red points, birds on migration in southern British Columbia). The points show values for wing plus tail measurements, characteristics which distinguish birds described as the *hooveri* subspecies of myrtle warblers. The line shows a LOESS smoothing function as implemented in R. The distribution of wing plus tail values of birds on migration is similar to the values observed in myrtle warblers at high latitudes

The combined measure of wing and tail lengths shows a strong latitudinal pattern: Above 60^o^N latitude, the wing/tail composite measure was, on average, much higher than at lower latitudes (Figure 2a). Our sample of myrtle warblers on migration has a distribution of wing plus tail lengths similar to those found at these high latitudes (Figure 2b) and is significantly different from those at lower latitudes (Table 2).

**Table 2:** Morphometric analysis of myrtle warblers sampled previously by Brelsford and Irwin (2009) and for the current study (from Vancouver, birds on migration in southern British Columbia). The birds from the current study are significantly different from those sampled at low latitudes for wing plus tail measures, metrics that best distinguish the *hooveri* subspecies. Bold *p*‐values indicate values below a critical value of .05

Location	Latitude (North)	Longitude (West)	*N*	Wing plus tail (mm)	*SD*	*p*‐value of *t* test compared to Vancouver (migration)
Anchorage, AK	61.16	149.75	23	133	2.8	.1322
Kluane, YT	61.03	138.41	6	135	3.3	.7375
Watson Lake, YT	60.00	129.05	10	133	3.2	.5389
Toad River, BC	58.88	125.40	11	129	2.8	**.0001**
Prophet River, AB	57.96	122.79	9	131	4.2	**.0094**
Fox Creek, AB	54.37	116.92	7	130	2.3	**.0013**
Slave Lake, AB	55.49	114.85	10	130	3.7	**.0010**
Rocky Mountain House, AB	52.42	115.01	8	130	2.8	**.0014**
Cold Lake, AB	54.73	110.06	9	129	3.4	**.0004**
Vancouver, BC	49.28	123.12	28	134	3.2	

**Figure 3:**
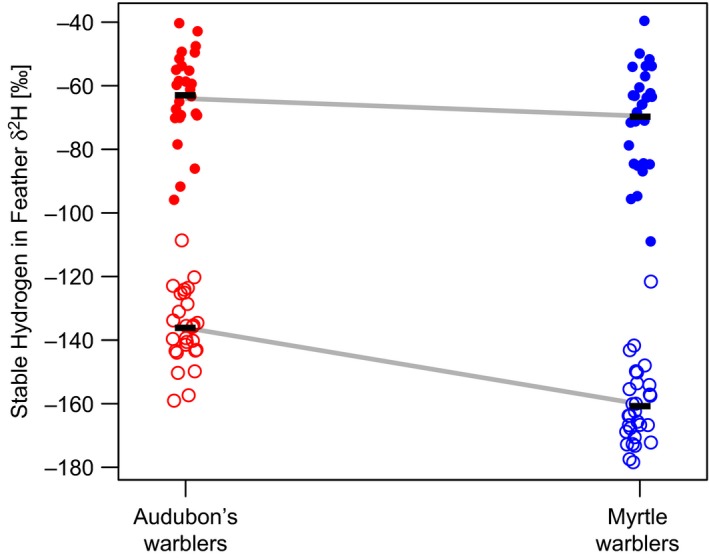
Stable hydrogen isotope values in feathers of Audubon's (red) and myrtle (blue) warblers captured on spring migration. Feathers grown during the prealternate molt (i.e., on the wintering grounds) are shown as filled circles. These feathers are grown further to the south and have higher hydrogen values. Feathers grown during the prebasic molt (i.e., on the previous breeding ground) are shown as open circles. These feathers are grown further north and have lower hydrogen values

**Figure 4:**
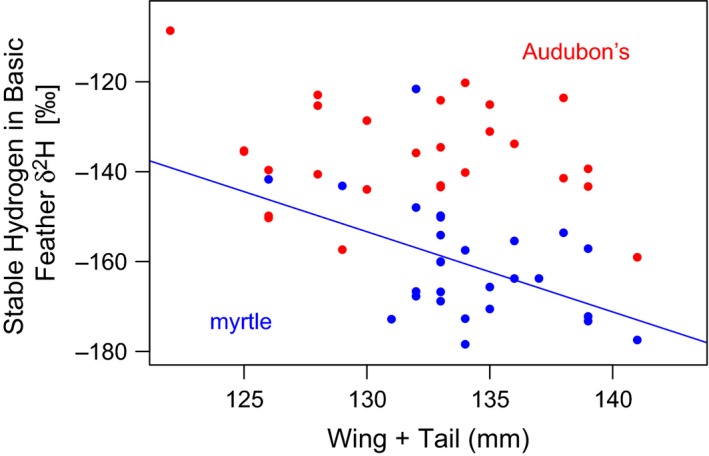
Stable hydrogen isotope values in feathers of Audubon's (red) and myrtle (blue) warblers captured on spring migration isotopes and wing plus tail measurements. There is a significant, negative relationship between wing plus tail and isotope values in myrtle warblers (*p *=* *.014) but not in Audubon's warblers (*p *=* *.384)

Patterns of stable hydrogen content differed between the species and varied based on when the feather was molted (Figure 3). For both species, feathers grown on the wintering grounds had a much higher proportion of deuterium as compared to those grown on the breeding grounds, consistent with previous studies in this species complex comparing analogous feathers (Toews et al., 2014). There was no significant difference in δ^2^Hf between the species for feathers grown during the prealternate molt (i.e., grown on the wintering grounds; mean of myrtle δ^2^Hf: −69.75‰ ± 15.8 *SD*; mean of Audubon's δ^2^Hf: −63.01‰ ± 13.9 *SD*;* t *=* *−1.67, *df* = 53, *p *=* *.10). By contrast, in the feathers grown during the prebasic molt (i.e., grown during the previous breeding season), myrtle warblers were highly depleted in δ^2^Hf as compared to Audubon's warblers, and this difference was significant (mean of myrtle δ^2^Hf −160.75‰ ± 12.4 *SD*; mean of Audubon's δ^2^Hf −136.16‰ ± 11.6 *SD*;* t *=* *−7.68, *df* = 54, *p *=* *3.3e^−10^). There was also a significant negative relationship between wing plus tail measurements and the raw isotope values of the basic in myrtle warblers (β = −1.78, *t *=* *−2.62, *df* = 26, *p *=* *.014; Figure 4). This relationship was not present in our sample of Audubon's warblers (*t *=* *−0.887, *df* = 25, *p *=* *.384; Figure 4).

**Figure 5:**
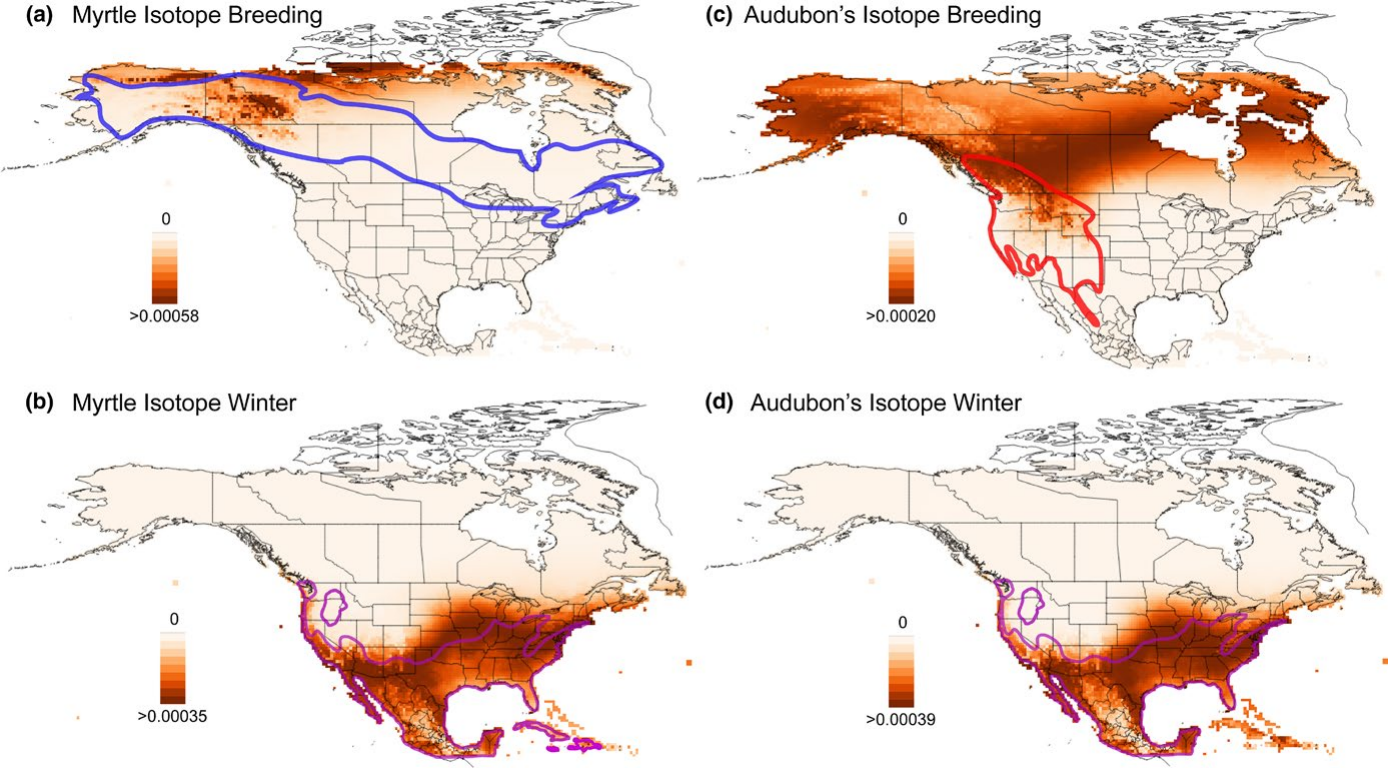
Likelihood surfaces of geographic origin of feathers based on their isotopic values, produced by IsoMAP. The likelihood surfaces are averaged by species and by where the feather was molted (i.e., on the breeding or wintering ground). We note that these maps are based on isotopes alone and include some regions that both warblers do not actually breed or winter. The extent of breeding ranges of myrtle and Audubon's warblers is shown in the blue and red outlines, respectively. The extent of wintering occurrence, outlined in purple, overlaps between the groups, although there are differences in local abundance in the different regions, as illustrated in Figure 1b and c

The geographic assignment likelihood surfaces are consistent with the interpretations from the raw isotope values. Assignment surfaces for alternate feathers—grown on the wintering ground—highlight similar regions between both myrtle and Audubon's warblers (Figure 5b and d), with high likelihood regions including the Pacific Coast, Baja California, the southwestern USA, as well as a large region throughout the eastern USA extending to the Atlantic Coast. The basic feathers, by contrast, differ strongly between the two species. For Audubon's warblers, regions of high likelihood are diffuse (Figure 5c) and include a wide band that extends from the Hudson Bay, through the boreal forest into the interior of British Columbia, and into Alaska. Regions of high likelihood for the basic myrtle warbler feathers are much more restricted and concentrated to latitudes above 60°N (Figure 5a). High likelihood regions include northern Alaska, the Yukon, and the high arctic of the Northwest Territories and Nunavut. This result is supportive of these myrtle warblers breeding in the postulated breeding range of *hooveri* (in northwestern Canada and Alaska) rather than in the range of *coronata* from Alberta to the east.

## 4 DISCUSSION

Here, we have used morphometrics and isotopes to link the wintering and breeding ranges of both western myrtle and Audubon's warblers. The central question we set out to address was whether the disjunct overwintering population of myrtle warblers along the Pacific Coast are predominately those that breed at high latitudes in northern British Columbia, the Yukon, and Alaska, rather than the alternative that myrtle warblers passing through southern British Columbia may largely be from breeding populations in Alberta or further east. Our data from birds sampled on spring migration are most consistent with the former interpretation. We expand on this, discuss some caveats, and draw more general conclusions below.

### 4.1 Wintering Pacific myrtle warblers breed at high latitudes

Myrtle warblers wintering along the Pacific Coast have been identified as a distinct subspecies—*Setophaga coronata hooveri*—primarily based on their long wings and tails (Bent, 1953; McGregor, 1899). In their original description, *hooveri* had average wing (76.7 mm) and tail lengths (58.4 mm) that were the longest recorded in populations of myrtle warblers (McGregor, 1899). At the time, these birds were presumed to be “breeding probably in British Columbia and Alaska” (McGregor, 1899). This was indirectly confirmed by studies of breeding birds in the north by Hubbard (1970). Hubbard (1970) sampled myrtle warblers from across their range and found only those at high latitudes had similarly long wings and tails. Our morphometric data from myrtle warblers migrating through southern British Columbia indicate they also have relatively long wings and tails, which is consistent with the description of breeding and wintering samples of *hooveri* (Figure 2 and Table 1).

A possible complication with this interpretation is that the large Pacific Coast myrtle warblers are not a wintering population drawn from a specific breeding location, but instead this region may represent a “filter” for large myrtle warblers attracted from multiple breeding populations. Moreover, the population differences in wing and tail lengths are not discrete and these groups have overlapping distributions. Our new isotopic data address the geographic origins of these birds more directly and our conclusions are consistent with the original presumption of McGregor (1899), connecting myrtle warblers in California to those in the high north.

The likelihood surfaces based on these isotopic data are diffuse in many cases—particularly for the alternate feathers and the basic feathers from Audubon's warblers—yet three patterns are relevant to this discussion. First, there is a region of high likelihood from the alternate feathers (grown in the winter), from both myrtle and Audubon's warblers, along the Pacific Coast. While there is also a large region of high likelihood in the southwest and eastern USA, based on the known wintering locations of these two taxa (e.g., the colored outlines in Figure 1b and d), this band of high likelihood is more likely due to the distribution of δ^2^H on the landscape as opposed to being reflective of their true overwintering locations (Toews et al., 2014). It is important to note that similar isotope values do not necessarily mean that feathers from the two groups were grown in the same location. However, at a broad scale, contrasting the two possible overwintering concentrations for myrtle warblers (e.g., Pacific Coast versus Gulf Coast), these results more strongly favor both Audubon's and myrtle warblers in our sample as wintering along the Pacific.

Second, for Audubon's warbler's basic feathers, the likelihood surface is very dispersed. However, it includes an area in central and eastern British Columbia where Audubon's are known to breed and is likely where these feathers were grown. Other regions to the north and east are beyond Audubon's warblers breeding range (Figure 5c) and thus make the origin of feathers from these locations very unlikely.

Finally, the average likelihood surface derived from basic feathers from myrtle warbler feathers is highly concentrated and is confined to latitudes north of 60°. Moreover, very few of the myrtle warbler likelihood surfaces have high likelihoods of originating from more southern latitudes, such as Alberta. The possible exceptions are the two myrtle warblers with smallest wing plus tail measures, which are also among the myrtle warblers with the highest isotope values (Figure 4). The likelihood surfaces also highlight regions further to the south (see DataDryad package for individual maps doi:10.5061/dryad.7ft57). This suggests that a small number of individuals in our migrant sample may have bred in lower latitude populations but that the large majority of these myrtle warblers instead bred at very high latitudes during the previous spring and summer. This is consistent with isotopic studies of Wilson's warblers (Kelly et al., 2002; Paxton, van Riper, Theimer, & Paxton, 2007; Paxton et al., 2013), with northern breeding populations similarly showing lower hydrogen isotope values. Like Audubon's and the non‐*hooveri* populations of myrtle warblers, Wilson's warblers consist of western and eastern genetically differentiated breeding populations that use distinct western and eastern migratory routes (Irwin, Irwin, & Smith, 2011; Paxton et al., 2013; Ruegg et al., 2014), presumably with a migratory divide between them.

Some caution is warranted in interpreting the likelihood surfaces of the geographic assignments from the myrtle basic feathers. This is because these feathers are highly depleted in deuterium. In fact, they are more depleted than most of the known‐location feathers included in the δ^2^Hf:δ^2^Hp transformation function utilized here (Hobson et al., 2012; Toews et al., 2014). Therefore, by estimating δ^2^Hp values from these feathers, we are necessarily extrapolating outside of the range of input values used in the model. While the precise geographic regions where these feathers were grown should therefore be treated with caution, it is clear given the extremely low levels of deuterium that these feathers were grown at very high latitudes, regions that are also depleted of deuterium.

### 4.2 The evolution of the migratory phenotype

These new data speak to three general conclusions that extend beyond migratory connectivity within the yellow‐rumped warbler species complex. First, these data are consistent with previous research in other migratory taxa that have found a correlation between migratory distance and wing length (Nowakowski, Szulc, & Remisiewicz, 2014). This is suggested by the significantly negative correlation between wing‐plus‐tail and deuterium‐depleted isotope values (Figure 4). This implies possible local adaptation of wing and tail morphology over a relatively small spatial scale, at least within the myrtle warblers. In great reed warblers (*Acrocephalus arundinaceus*), as in other avian taxa, wing length has been shown to vary positively with migration distance (Tarka et al., 2010). At a broader scale, across different groups within the yellow‐rumped warbler species complex, there is evidence that wing shape differs predictably between migratory and sedentary populations, consistent with what would be expected given the selection pressures acting on the different life history strategies (Milá, Wayne, & Smith, 2008). The fact that myrtle warblers breeding at high latitudes have longer wings is consistent with these populations being morphologically adapted to a distinct migratory route.

A second conclusion from our study is that there may be a migratory divide within the range of myrtle warblers. A migratory divide was previously postulated to exist between myrtle and Audubon's warblers (e.g., Rohwer & Irwin, 2011), since Audubon's warblers winter almost entirely along the Pacific Coast of the USA and Mexico whereas most myrtle warblers winter in the southeastern USA. However, this conclusion was complicated by the fact that some myrtle warblers winter on the Pacific Coast. A previous isotopic analysis of the hybrid zone between myrtle and Audubon's warblers by Toews et al. (2014) found that birds from opposite sides of their hybrid zone in the Rocky Mountains winter in isotopically distinct locations, consistent with the suggestion that the hybrid zone generally aligns with a migratory divide. The pattern among hybrids was less clear, however, with most having wintering isotopic values more similar to myrtle warblers, but some having values more similar to Audubon's warblers. The overlap in values between the allopatric groups and the similarity in hydrogen isotopic values between the Pacific Coast and the southeastern USA make it difficult to map the wintering locations of hybrids with precision. Regardless, the current results raise the possibility that Audubon's warblers and the *hooveri* form of myrtle warblers have similar migratory routes along the Pacific coast, and each forms a migratory divide with the *coronata* form of myrtle warbler in different regions.

Any divide within myrtle warblers likely occurs between genetically similar populations: Several myrtle warblers sampled from Alaska were included in genomic analyses of the species complex (Brelsford, Milá, & Irwin, 2011; Toews, Brelsford, Grossen, Mila, & Irwin, 2016). These individuals from Alaska—within the presumed range of *hooveri*—appear very similar genetically to other myrtle warblers sampled from across the range (Brelsford et al., 2011; Toews et al., 2016). However, these previous studies included only limited sampling of possible *hooveri* individuals. Moreover, important genes for migration may be restricted in the genome and therefore possibly not assayed by AFLP approach used by Brelsford et al. (2011) or the reduced genome sequencing approach employed by Toews et al. (2016). Additional genomic study of northern myrtle warbler populations will be useful in addressing any association between migration and more subtle patterns of genetic differentiation within the group.

Finally, these data are consistent with the evolution of a novel overwintering location within myrtle warblers along the Pacific Coast. Myrtle warblers are one of the few eastern boreal warblers that have a breeding range that extends far into the northwest as well as a disjunct Pacific Coast wintering population. Our interpretation is that the evolution of this wintering range and migration route along the Pacific Coast may have facilitated the breeding expansion of myrtle warblers into the northwest.

We suggest two possible ways that an eastern warbler with an eastern migratory route could evolve a west‐coast migratory route: through within‐population evolution of the migratory program, or alternatively through introgression of alleles for the western route from other taxa. Hybridization with western‐migrating Audubon's warblers may have introduced alleles that predispose individuals toward using the western route. Audubon's and myrtle warblers were likely separated for an extensive period during Pleistocene glaciations. If Audubon's warblers were already well adapted for wintering in California, the eastern‐adapted myrtle warbler may have acquired those western‐adapted alleles through hybridization. This remains a speculative idea at this time, but introgression is increasingly viewed as a common way that populations acquire advantageous alleles and may operate on a faster timescale than novel mutation.

### 4.3 Other taxa and next steps

Other species have similarly distinct and separate migratory routes and/or overwintering populations and would be fascinating to examine. Palm warblers (*Setophaga palmarum*), for instance, winter mostly in Florida and the Caribbean, but also consistently along the coast of California, Oregon, and Washington. Distinct western and eastern subspecies have also been described within palm warblers (Wilson, 1996). Almost all palm warblers observed on the west coast are from the western subspecies (Wilson, 1996), but this subspecies also winters in large numbers in the southeast, suggesting a possible migratory divide within the western subspecies.

Understanding why several species have such clearly disjunct wintering populations will be useful in understanding the evolution of novel overwintering sites and migration routes. We suggest that the evolution of new wintering ranges and migration routes might generally facilitate breeding range expansions. Whether this is a general phenomenon will require additional data from this and other species groups.

## CONFLICT OF INTEREST

None declared.

## AUTHOR CONTRIBUTIONS

All authors worked together to conceive the idea for the project. JH collected the samples for the analysis. DPT analyzed the data and wrote the original draft of the manuscript. All authors contributed to the writing and editing of the manuscript.
