# Supplementary material for: Linking the wintering and breeding grounds of warblers along the Pacific Flyway
Source: Ecol Evol. 2017 Jul 18;7(17):6649–58. doi: 10.1002/ece3.3222 (PMC5587466; doi:10.1002/ece3.3222)
Supplement: Supplementary file 1 [file ECE3-7-6649-s001.docx]

**Supporting Information from:**

Linking the wintering and breeding grounds of migrating warblers along the Pacific Flyway

David P. L. Toews^1,2^, Julian Heavside^1^, Darren E. Irwin^1^

**Supplementary Table 1:** Standard information from Cornell Isotope Laboratory

| Day | mean CBS (‰) | SD CBS (‰) | mean KHS (‰) | SD KHS (‰) | mean keratin (‰) | SD keratin (‰) |
| --- | --- | --- | --- | --- | --- | --- |
| Jan-15-2015 | -196.91 | 2.03 | -54.19 | 3.18 | -115.26 | 1.00 |
| Jan-16-2015 | -196.91 | 2.38 | -54.19 | 3.27 | -111.90 | 1.16 |
